# Focal segmental glomerulosclerosis lesion associated with inhibition of tyrosine kinases by lenvatinib: a case report

**DOI:** 10.1186/s12882-018-1074-3

**Published:** 2018-10-19

**Authors:** Yoshitaka Furuto, Hirotsugu Hashimoto, Akio Namikawa, Haruki Outi, Hiroko Takahashi, Hajime Horiuti, Kazuho Honda, Yuko Shibuya

**Affiliations:** 1 Department of Hypertension and Nephrology, NTT Medical Centre Tokyo, 5-9-22, Higasi-Gotanda, Shinagawa-ku, Tokyo, 141-8625 Japan; 2 Department of Diagnostic Pathology, NTT Medical Centre Tokyo, 5-9-22, Higasi-Gotanda, Shinagawa-ku, Tokyo, 141-8625 Japan; 3 Department of Microscopic Anatomy, Showa University Hospital, 1-5-8, Hatanodai, Shinagawa-ku, Tokyo, 142-8666 Japan

**Keywords:** Lenvatinib, Tyrosine kinase inhibitors, Focal segmental glomerulosclerosis, Thyroid cancer

## Abstract

**Background:**

Lenvatinib is a tyrosine kinase inhibitor with novel binding ability. It is considered the standard of care for metastatic thyroid cancer; moreover, whether it is indicated for other malignant tumors has been examined. Lenvatinib increases the risk of kidney injury in some patients. In comparison with sorafenib, which is a conventional tyrosine kinase inhibitor (TKI), lenvatinib results in more side effects, including hypertension and proteinuria. We describe a case of secondary focal segmental glomerulosclerosis (FSGS) that developed following treatment of metastatic thyroid cancer with lenvatinib and reviewed the mechanisms of renal impairment.

**Case presentation:**

We describe a patient with metastatic thyroid cancer who developed hypertension, nephrotic syndrome, and acute kidney injury after 3 months of lenvatinib treatment. Renal biopsy results revealed that 7 of 16 glomeruli indicated complete hyalinization, and that the glomeruli with incomplete hyalinization showed FSGS due to a vascular endothelial disorder and podocyte damage, which seemed to have been induced by lenvatinib treatment. These findings were similar to those of renal impairment treated with conventional TKIs. Although lenvatinib treatment was discontinued, up to 15 months were required to achieve remission of proteinuria, thus leading to chronic kidney disease with hyalinized lesions.

**Conclusions:**

To the best of our knowledge, this is the first reported case of secondary FSGS by lenvatinib treatment. Renal impairment treated with TKIs is commonly associated with minimal change nephrotic syndrome/FSGS findings, and it is suggested that renal involvement with TKI is different from that with the vascular endothelial growth factor ligand. Overexpression of c-mip due to TKI causes disorders such as podocyte dysregulation and promotion of apoptosis, which cause FSGS. Lenvatinib may result in FSGS by a similar mechanism with another TKI and could cause irreversible renal impairment; therefore caution must be used. It is essential to monitor blood pressure, urinary findings, and the renal function.

## Background

Lenvatinib is an oral tyrosine kinase inhibitor with novel binding ability and selective inhibition against receptor-type tyrosine kinase involved in tumor angiogenesis and malignant tumor transformation [1–3]. The target molecules of lenvatinib are vascular endothelial growth factor (VEGF) receptors (VEGFRs) 1–3, fibroblast growth factor receptors (FGFR)1–4, platelet-derived factor receptor-α (PDGFRα), RET (rearranged during transfection), and KIT proto-oncogenes [1–3]. Because lenvatinib has greater inhibitory activity against VEGFRs and inhibits FGFR more than other VEGF inhibitors [1–3], it is expected to have therapeutic effects on patients who have developed resistance to other VEGF inhibitors [4]. Moreover, whether lenvatinib is indicated for the treatment of various malignant tumors has been examined [5]. For thyroid cancer, neovascularization is necessary for tumor growth and metastasis. Because the expression of angiogenesis factors, such as VEGF, tyrosine kinase receptors are enhanced [6]. Therefore, it is expected that tumor regression can be achieved by using tyrosine kinase inhibitors (TKIs) such as lenvatinib, sorafenib, and sunitinib.

Tyrosine kinase inhibitors can be classified as type I to type V according to the binding site of the target kinase and the conformation of kinases on inhibitor binding. Most TKIs are type I or type II. An analysis of the co-crystal structure using X-rays revealed that lenvatinib has a novel ability to bind to VEGFR2 (type V). A kinetic analysis demonstrated that lenvatinib binds to the target molecule immediately and strongly inhibits kinases, and it has been speculated that its novel binding ability may contribute to these actions [7].

In Japan, lenvatinib was recently approved as a therapeutic drug for the treatment of unresectable thyroid cancer [4]. In 2018, it was realized that this drug was indicated for unresectable hepatocellular carcinoma [8]. Lenvatinib is considered the standard of care for metastatic thyroid cancer because of its inhibitory effectiveness and because it does not require dose adjustments based on renal function or therapeutic drug monitoring (TDM) [9].

Lenvatinib is a TKI with novel binding ability; however, it is associated with more side effects, including hypertension and proteinuria, compared with sorafenib, which targets VEGFRs 1–3, RET, RAF, and PDGFRβ; in addition, lenvatinib has been associated with acute kidney injury in some patients [4, 10, 11]. Renal impairment caused by conventional TKIs commonly involves minimal change nephrotic syndrome/focal segmental glomerulosclerosis (FSGS)-like lesions, which are caused by impaired podocytes [12].

We describe a case of secondary FSGS that developed following treatment of metastatic thyroid cancer with lenvatinib. We also discuss the possible pathomechanisms by which lenvatinib likely induced glomerular damage.

## Case presentation

A 79-year-old woman initially presented with diabetes in 2008. Her HbA_1c_ level was 8.8%, and treatment with an oral hypoglycemic agent was initiated. At that time, the patient also reported swelling on the anterior surface of her neck in the area of the thyroid gland; therefore, she sought consultation at an ambulatory otolaryngology clinic. She was diagnosed with papillary thyroid carcinoma with metastasis to the right cervical lymph nodes. In 2011, a pulmonary tumor was detected; it was resected via thoracoscopy and a diagnosis of metastatic thyroid cancer was confirmed. In December 2012, her serum creatinine level was 0.57 mg/dL, with no evidence of proteinuria. In 2013, the patient underwent resection of the right lobe of the thyroid gland, including bilateral dissection of the paratracheal lymph nodes and the right parotid lymph node.

In January 2016, the patient experienced exacerbation of her unresectable thyroid cancer; therefore, oral administration of 10 mg lenvatinib was initiated.

**Fig. 1:**
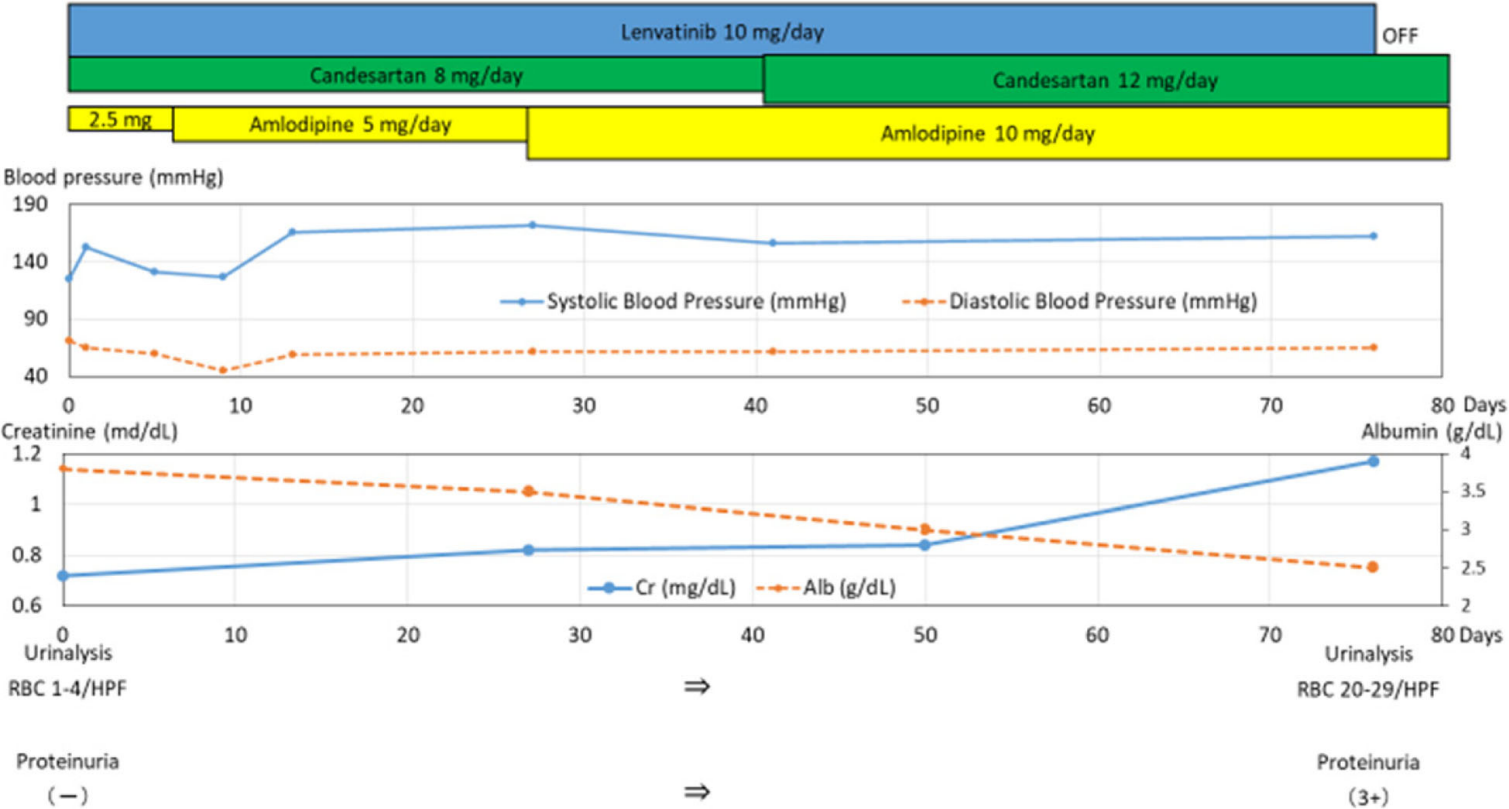
Graph showing the findings before and after the introduction of lenvatinib. The upper section depicts the treatment with lenvatinib and an antihypertensive agent. The antihypertensive agent was gradually increased. The middle section shows the course of blood pressure, serum creatinine, and serum albumin. Despite the increase in the quantity of the antihypertensive agent, blood pressure and serum creatinine increased, and serum albumin decreased. The lower section shows urinalysis results. Before lenvatinib administration, urinalysis results were normal. After lenvatinib administration, the urine quantitative analysis revealed the presence of red blood cells (20–29/HPF) and proteinuria (3+)

The findings of the pretreatment laboratory assessment were as follows: normal blood pressure (118–132/64–77 mmHg); creatinine (Cr), 0 .72 mg/dL; and albumin (Alb), 3.8 g/dL, respectively, and her estimated glomerular filtration rate (eGFR) was 58 mL/min/1.73 m^2^. In addition, the urine sample test showed negative results for red blood cells (1–4/HPF), and the urine qualitative analysis showed negative results for protein. However, after initiation of treatment (Fig. 1), the patient developed hypertension that required treatment with candesartan (8 mg/day). By February 2016, her Cr level had increased to 0.82 mg/dL, and her eGFR and Alb levels had decreased to 51 mL/min/1.73 m^2^ and 3.5 g/dL, respectively. She also developed hypertension (blood pressure, 140–170/60–70 mmHg). Based on these findings, we added a daily dose of amlodipine (5 mg/day) to her treatment; thereafter, the dose was increased to 10 mg/day. By March 2016, her Cr level continued to increase to 0.84 mg/dL, and her eGFR and Alb level continued to decrease (49 mL/min/1.73 m^2^ and 3.0 g/dL, respectively). Her blood pressure increased to 150/60 mmHg, and the candesartan dose was increased to 12 mg/day. However, she developed lower limb edema. In April 2016, she was diagnosed with acute kidney injury and nephrotic syndrome. Development of generalized edema and weight gain were noted, her Cr level increased to 1.17 mg/dL, and her eGFR decreased to 34 mL/min/1.73 m^2^. Measurements of other relevant parameters were as follows: total protein (TP), 5.1 mg/dL; Alb, 2.5 mg/dL; total creatinine (TC), 329 mg/dL; low-density lipoprotein (LDL), 204 mg/dL; and urinary protein, 11.78 g/gCr. The patient was referred to our institution for further evaluation and treatment.

### Clinical presentation on admission

On admission, the patient’s medications included lenvatinib (10 mg), glimepiride (0.5 mg), pioglitazone (915 mg), alogliptin (925 mg), candesartan (12 mg), and amlodipine (10 mg). On physical examination, her height was 155 cm, weight was 55 kg (usual weight, 44 kg), body mass index was 21.4 kg/m^2^, blood pressure was 142/60 mmHg, heart rate was 72 beats/min, temperature was 36.2 °C, and respiratory rate was 20 breaths/min. A surgical scar from her thyroidectomy was visible on the anterior aspect of her neck. In addition, bilateral edema of her lower limbs was evident. Significant medical history included a uterine myoma, appendicitis, and a fundal hemorrhage due to diabetic retinopathy at the ages of 50, 51, and 77 years, respectively. Her family history was negative, and she had no known allergies. The patient was a non-smoker and only consumed alcohol socially. Her urine and blood laboratory data are summarized in Table 1. Large quantities of urine protein and urine occult bleeding, anemia, renal function disorder, hypoalbuminemia, and hypercholesterolemia were observed. Urinary Bence-Jones proteins, increased ferritin, hypergammopathy, low-complement blood symptoms, and ANCA and ds-DNA antibodies were not observed, however.

**Table 1:** Laboratory data on admission

Urinalysis		Biochemistry/Immunology			
Protein Occult blood Red blood cell Protein content Bence-Jones protein	3+ 3+ 20–29/HPF 11.78 g/gCr (−)	TP Alb UA BUN Cr eGFR TB AST ALT ALP γ-GT ChE LDH CK Na K Cl cCa IP CRP	5.1 g/dL 2.5 g/dL 4.5 mg/dL 22.3 mg/dL 1.16 mg/dL 35 mL/min/1.73 m^2^ 0.4 mg/dL 22 IU/L 14 IU/L 290 IU/L 170 IU/L 241 IU/L 312 IU/L 326 IU/L 140 mEq/L 5.1 mEq/L 107 mEq/L 8.4 mg/dL 4.7 mg/dL 0.7 mg/dL	TC LDL HbA_1c_ TSAT Ferritin HANP BNP IgG IgA IgM C3 C4 CH50 Anti-dsDNA-Ab Anti-MPO-ANCA Anti-PR3-ANCA	329 mg/dL 204 mg/dL 5.7% 25% 41 ng/mL 240.7 pg/mL 116.9 pg/mL 754 mg/dL 219 mg/dL 107 mg/dL 117 mg/dL 32 mg/dL 45 U/mL (−) (−) (−)
Complete blood cell count					
White blood cell Neutrophil Lymphocyte Monocyte Eosinophil Red blood cell Hemoglobin Hematocrit Platelet	6300/μL 80.7% 13.6% 4.4% 0.5% 299 × 10^4^/μL 8.6 g/dL 26.5% 19.6 × 10^4^ μL				

*cCa* corrected calcium

Multiple pulmonary metastases were observable on computed tomography (CT) imaging. Although there was no evidence of malformation of the kidneys, generalized edema and thoraco-abdominal fluid were observed.

### Treatment course

Considering the development of elevated blood pressure and nephrotic syndrome after administration of lenvatinib, drug-induced nephrotic syndrome was suspected as the primary clinical diagnosis. Therefore, lenvatinib treatment was discontinued.

The patient’s diabetes was controlled well with the use of oral hypoglycemic agents (HbA_1c_ of 5.7%). However, her blood pressure remained high at 140–145/50–60 mmHg despite treatment with oral antihypertensive agents. Because of her history of diabetic retinal hemorrhage, papillary thyroid carcinoma with relatively new onset of distant metastasis, hematuria, low selectivity index (0.247), information based on the negative findings of hypocomplementemia, absence of MPO-ANCA, PR3-ANCA, ds-DNA antibodies, we thought that minimal change nephrotic syndrome (MCNS), membranoproliferative glomerulonephritis (MPGN), and rapidly progressive glomerulonephritis (RPGN) would not be correct diagnoses.

Because it is necessary to differentiate FSGS from diabetes nephrosis syndrome and secondary membranous nephropathy due to a malignant tumor, and because most TKIs are type I or type II and lenvatinib has novel binding ability (type V), we performed a renal biopsy.

**Fig. 2:**
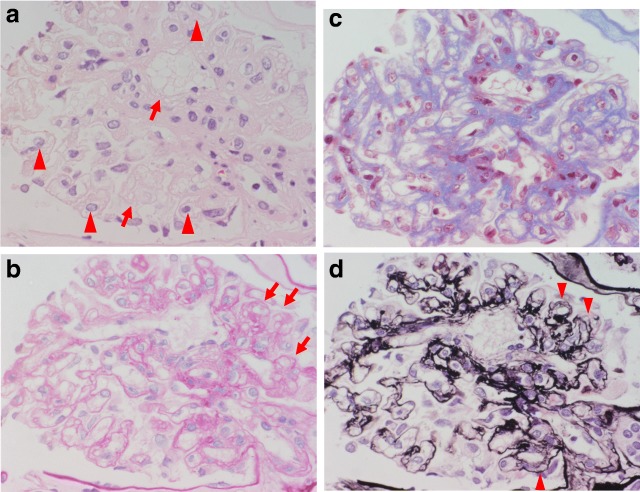
Renal histopathological findings of light microscopy. Results of the renal biopsy using light microscopy: **a**, hematoxylin-eosin stain; **b**, periodic acid-Schiff stain; **c**, Masson trichrome stain; and d, periodic acid-Methenamine-silver stain. All images were magnified 60×. Seven of 16 glomeruli indicated complete hyalinization, and the glomeruli with incomplete hyalinization showed partial glomerular collapse (arrow in **b**). The diagnosis was FSGS because the glomeruli showed lobular and segmental expansion. Vacuolar degeneration of podocytes (arrow in **a**) and enlarged endothelial cells with a thickened loop were observed, suggesting endothelial injury (arrow head in **a**). Thrombotic microangiopathy was absent. The mesangial matrix slightly increased, but the number of mesangial cells did not increase (**b**, **c**). Periodic acid methenamine silver (PAM) staining showed mesangial interposition-like changes and a duplicated basal membrane (arrow head in **d**). The endarterium is slightly thickened and the arterioles show partial hyaline consolidation

Renal biopsy using light microscopy (Fig. 2a-d) revealed that 7 of 16 glomeruli had complete hyalinization, and that glomeruli with incomplete hyalinization showed partial glomerular collapse (arrow in Fig. 2b); FSGS was confirmed because the glomeruli showed lobular and segmental expansion. Vacuolar degeneration of the podocytes (see arrow in Fig. 2a) and enlarged endothelial cells with a thickened loop were evident, suggesting endothelial injury (arrow head in Fig. 2a). Thrombotic microangiopathy was absent. The mesangial matrix slightly increased, but the mesangial cells did not increase (Fig. 2b, c). Periodic acid methenamine silver (PAM) staining showed mesangial interposition-like changes and a duplicated basal membrane (arrow head in Fig. 2d). The endarterium was slightly thickened and the arterioles showed partial hyaline consolidation.

**Fig. 3:**
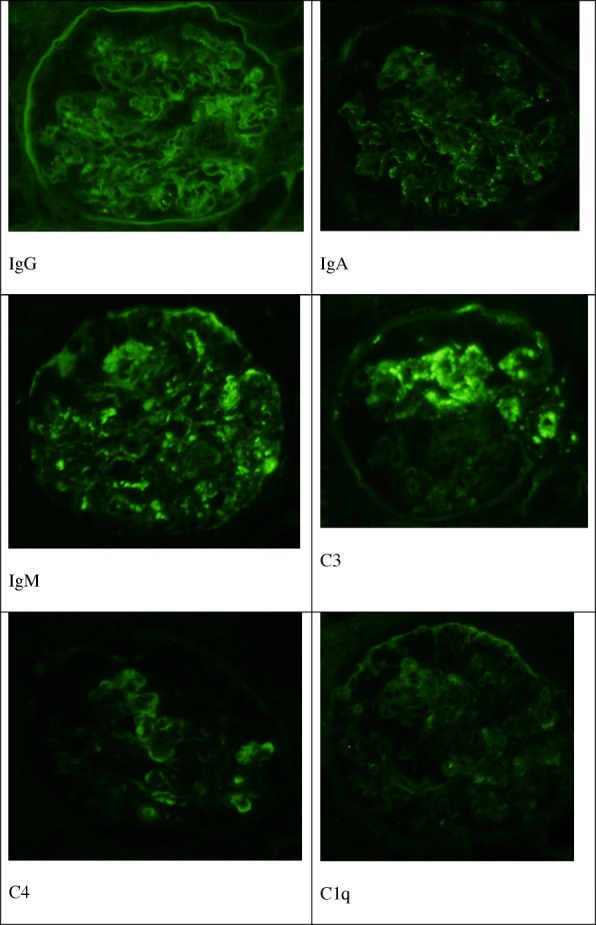
Fluorescent antibody screening. Immunofluorescence of IgG showed nonspecific staining, no linear pattern, and negative results. Only IgA, IgM, C3, and C4 were granular and slightly positive in the mesangial areas, and no staining of the loop wall was shown. Negative C1q was observed

Immunofluorescence (Fig. 3) of the IgG showed nonspecific staining, no linear pattern, and negative results. Only IgA, IgM, C3, and C4 were granular and slightly positive in the mesangial areas, and there was no staining of the loop wall. C1q was negative.

**Fig. 4:**
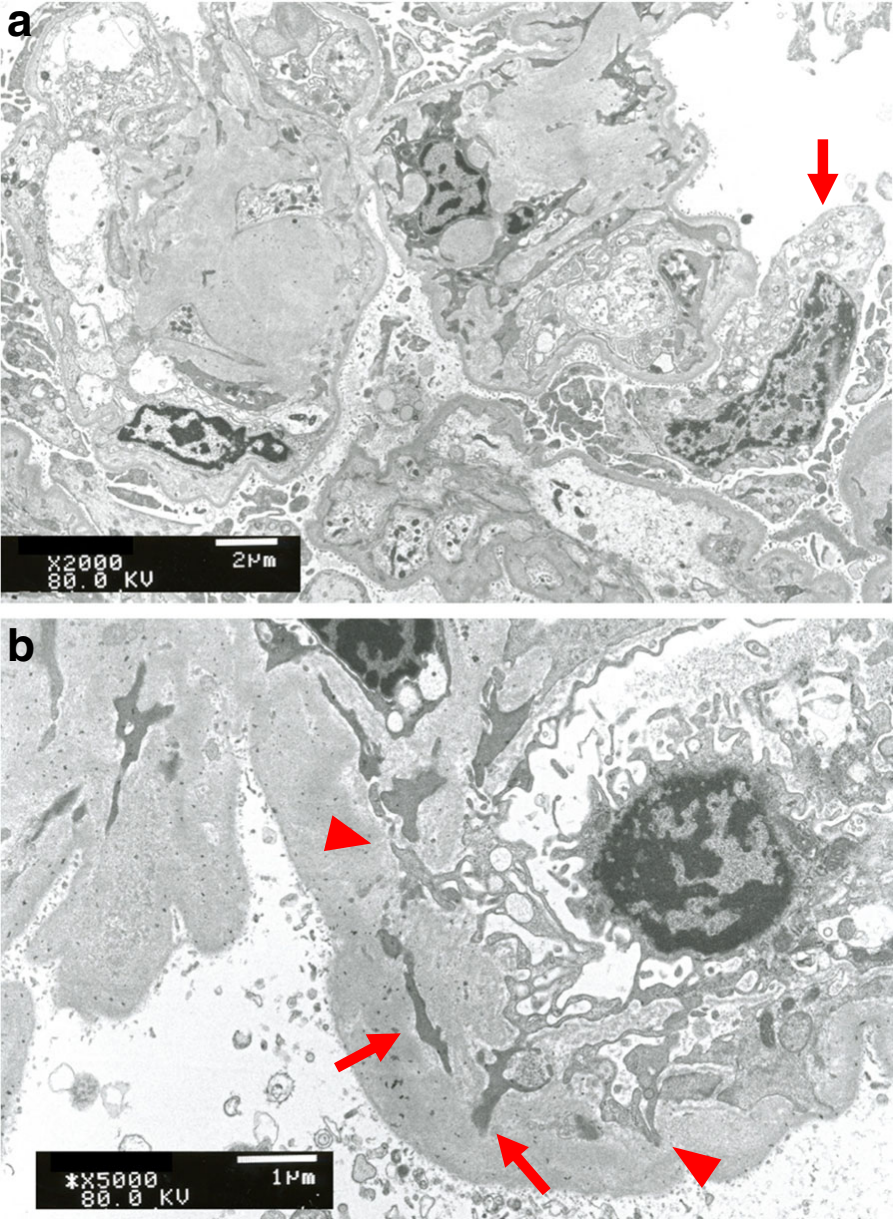
Electron microscope image (a: × 2000; b: × 5000). Vacuolar degeneration of podocytes (arrow in **a**) was observed using electron microscopy (**a**, **b**). The loops were thickened. A duplicated basal membrane was observed. Mesangial interposition-like changes were found (arrow in **b**). Endothelial cells invaded the basal membrane (arrow head in **b**)

Vacuolar degeneration of podocytes (arrow in Fig. 4a) was observed on electron microscopy (Fig. 4a, b). The loops were thickened and a duplicated basal membrane was observed. Mesangial interposition-like changes were found (arrow in Fig. 4b), and endothelial cells invaded the basal membrane (arrow head in Fig. 4b).

Electron-dense deposits suggesting immune complexes were not observed. Foot process effacement was almost 60–70%. It suggested that this is a case of podocytopathy and not a secondary effect of hypertensive/hyperfiltration injury.

The mesangial matrix slightly increased, but there was no nodular glomerulosclerosis such as a Kimmelstiel-Wilson lesion or IgG immunofluorescence staining with a linear pattern along the basement membrane, thereby suggesting that the diagnosis was not advanced stage disease that could lead to diabetes nephrosis syndrome. The glomeruli showed lobular expansion, but mesangial cells were not increased and an electron-dense deposit was not observed; therefore, the diagnosis was not MPGN.

With regard to morphologic variants of FSGS based on the Columbia classification, the collapsing variant of FSGS is diagnosed because of the presence of collapsed glomeruli.

Treatment included the discontinuation of lenvatinib, dietary modifications, and the use of palliative diuretics. The patient’s weight decreased over the course of the subsequent 2 weeks (loss of 8.7 kg from admission weight) but showed improvement in the edema of her lower limbs. Glycemic control was achieved. Because nephrotic syndrome was improved after discontinuation of lenvatinib therapy, and because diabetic changes in the kidneys including increased thickness of the mesangial matrix were mild, we confirmed a diagnosis of secondary FSGS caused by lenvatinib.

The patient was discharged on day 11 after admission. Although her renal function status did not improve from that on admission (Cr, 1.16 mg/dL; eGFR, 35 mL/min/1.73 m^2^) to that at discharge (Cr, 1.17 mg/dL; eGFR, 34 mL/min/1.73 m^2^), her proteinuria did improve from 11.8 g/day on admission to 5.4 g/day at discharge. She was followed-up on an outpatient basis, and her proteinuria continued to gradually decrease over time. In July 2017, her Cr level was 1.04 mg/dL and eGFR was 39 mL/min/1.73 m^2^, with complete remission of urinary protein (qualitative urinary protein, 116 mg/gCr) and no evidence of hematuria. A period of 15 months was required to achieve complete remission after discontinuation of lenvatinib treatment. Chronic kidney disease (CKD; stage G3bA1) was diagnosed. Regular follow-up examinations are performed at our outpatient clinic.

## Discussion and conclusions

This is the first report of renal histopathological findings associated with secondary FSGS due to a vascular endothelial disorder and podocyte damage induced by lenvatinib treatment. Nephrotic syndrome developed at approximately 3 months following initiation of lenvatinib treatment for metastatic thyroid cancer in our patient. The development of lesions with complete hyalinization caused irreversible renal failure.

Results of the international cooperative phase III trials [4, 13, 14] identified the following adverse side effects as risk factors for lenvatinib treatment: hypertension (incidence rate, 67.8%); urinary protein (32.6%); hematuria (5%); and nephrotic syndrome (0.4%; a single case). Of these, hypertension (10%) and urinary protein (10%) were included in the main toxicity criteria with a grade ≥ 3. Therefore, it seems reasonable to consider that dose adjustment might be necessary to lower the risk of toxicity [9, 14].

The period between initiation of lenvatinib treatment and the advent of renal impairment was 43 (range, 5–799) days, with a mean of 43 (range, 13–560) days in Japan [4, 13–15].

Side effects of lenvatinib can be observed at any time following lenvatinib initiation [12], and these side effects are caused by the effects of lenvatinib on VEGF inhibition [4, 13–15]. There might be an opportunity to perform dose modification to lower the risk of these renal events; however, it takes an average of 8.8 weeks to resolve proteinuria after dose modification of lenvatinib [14].

Effects of VEGF inhibition on blood pressure should be considered. VEGF regulates blood pressure through its effect on vascular tone; specifically, VEGF inhibitors suppress nitric oxide (NO) and phospholipase A2 activation, which decrease the production of prostacyclin (PGI_2_) and increase the release of endothelin-1 (ET-1) as a vasoexcitor, thus causing vasoconstriction. Oxidative stress due to VEGF inhibition results in endothelial cell injury, a decrease in microvessel network density, and increased vascular resistance. Furthermore, the reduction of NO leads to increased vascular volume via reabsorption of sodium. Increased vascular resistance and increased vascular volume due to these mechanisms result in hypertension [15–17].

The use of VEGF inhibitors is also associated with the risk of proteinuria, and the prevalence of hypertension is higher among patients with proteinuria [18]. It has been proposed that proteinuria develops due to VEGF inhibition-derived changes in glomerular architecture and impairments in filtration function [19].

VEGF is produced by podocytes and binds to VEGFR2, which is expressed by endothelial cells [20]. The interactions of podocyte VEGF and glomerular VEGFR regulate endothelial function and glomerular vascular permeability [21, 22]. This impairs the regulation of the tight junction, allowing an increased passage of protein that impairs normal glomerular function [19, 21, 23, 24]. The risk of proteinuria due to the use of VEGF inhibitors has been reported to be higher among Asian populations [25]. In our case study, renal biopsy findings were indicative of secondary FSGS resulting from damage to the vascular endothelium and podocytes. The development of nephrotic syndrome secondary to the use of VEGF inhibitors has been reported [21, 26, 27]. Furthermore, a case report described the development of thrombotic microangiopathy that resulted in nephrotic syndrome and secondary FSGS, similar to our case, in a patient treated with the TKI sunitinib [27].

Although the cause of FSGS associated with the use of VEGF inhibitors has not been clearly determined, it is known that vascular endothelial damage occurs locally at the site of podocyte loss [28–30]. FSGS is also a typical glomerular change among patients with eclampsia who develop nephrotic syndrome [31]. In this clinical population, the placenta promotes the expression and secretion of soluble feline McDonough sarcoma virus (fms)-like tyrosine kinase-1 (sFlt1) that inhibits the bioactivity of VEGF, which is considered the cause of vascular endothelial damage. Because VEGF inhibitors or adenoviruses that express sFlt1 are associated with the onset of eclampsia, there may be an association between VEGF inhibition and the process of vascular endothelial damage resulting in FSGS [21, 32].

Among patients treated with bevacizumab as a VEGF ligand and sunitinib as a TKI, the following renal pathologies have been reported: FSGS [17, 27, 33]; thrombotic microangiopathy (TMA) [17, 21, 27, 34, 35]; crescentic glomerulonephritis [17, 36]; minimal change nephropathy [17, 37]: tubulointerstitial nephritis or tubular necrosis [17, 37–39]; cryoglobulin-related glomerulonephritis [40]; glomerular collapse [41]; proliferative immune-complex glomerulonephritis [42, 43]; and TMA and IgA deposits with glomerular collapse [32]. Currently, it is not clear whether the changes in renal structure and function might be reversible. In patients with anti-VEGF-induced TMA, FSGS and MCNS, podocytes are mostly depleted with VEGFs [12]. VEGF inhibition is believed to be the underlying mechanism leading to the development of podocyte and endothelial cell damage [17, 25, 34].

VEGF ligands (anti-VEGF monoclonal antibody) target VEGFRs 1, 2, and 3, whereas TKIs interfere with the activity of VEGFR and other growth factor receptors, such as PDGF receptors, stem cell factors, FMS-like tyrosine kinase-3, b-raf, and Bcl-Abl. Therefore, they are commonly called multitargeted TKIs [32].

Patients receiving receptor TKIs mainly develop MCNS/FSGS-like lesions, whereas TMA complicates VEGF ligand therapy [12, 44]. Therefore, it is suggested that renal impairment can be classified into two types of VEGF-targeted therapy. It is also suggested that the renal involvement of TKI is different from that of VEGF ligands.

With TMA, c-mip was not detected, and an abundance of RelA, which is part of the NK-κB family, was observed. However, in MCNS/FSGS-like cases, an abundance of c-mip was observed and a few RelA were detected.

TKIs inhibit RelA activity and promote c-mip expression [12]. Similarly, in TMA, KI-67 was not observed and a normal amount of synaptopodin-like control was confirmed. In MCNS/collapsing FSGS cases, an abundance of KI-67 was found and no synaptopodin was detected. More often, podocytopathies are secondary to TKIs than to VEGF ligands [44]. Furthermore, the finding that the TKI sorafenib induces impressive cytoskeleton changes in cultured podocytes suggests that this class of therapy has direct effects on podocytes [12].

Uncontrolled NF-κB has an important pathophysiologic role in impaired podocytes. Inhibition of NK-κB activity by TKIs leads to overexpression of c-mip [45]. Additionally, it has been reported that overexpression of c-mip causes impaired podocytes, such as podocyte dysregulation and the promotion of apoptosis leading to MCNS/FSGS, resulting in progressive FSGS [46–48]. Histologic findings of our case have implied that FSGS due to lenvatinib is associated with these mechanisms.

To the best of our knowledge, kidney biopsy results of patients with renal dysfunction due to the use of lenvatinib have not been previously reported despite previous reports of secondary hypertension, proteinuria, and acute nephrotic syndrome. Based on the current evidence, patients treated with TKIs, including lenvatinib, should undergo blood pressure and urinary protein screening for early detection of hypertension and, as needed, aggressive administration of antihypertensive agents. Regarding hypertension treatment, angiotensin-converting enzyme inhibitors and renin-angiotensin system inhibitors have been shown to be effective for lowering the risk of vascular endothelial damage through their effects on expanding efferent arterioles, reducing glomerular pressure, and decreasing proteinuria via the intercellular spaces of the glomerular podocytes. Therefore, blood pressure control using these antihypertensive agents is important. Moreover, lowering the dose of TKIs, or their withdrawal and discontinuation, should be considered for patients who develop uncontrolled hypertension or other side effects [14]. Depending on the clinical symptoms and severity of glomerular disease, the following schedule of lenvatinib withdrawal is suggested: 20, 14, 10, 8, or 4 mg once per day [9].

The criteria for withdrawal, reduction, and discontinuation of lenvatinib are summarized in Table 2 [9]. The package insert for lenvatinib recommends reduction or withdrawal of the drug for patients who develop proteinuria because the incidence of proteinuria is considered dose-related and variable for different VEGF inhibitors [49–51]. Age is also a risk factor for renal damage secondary to the use of VEGF inhibitors, with higher risk found among elderly patients [52].

**Table 2:** Criteria for withdrawal, reduction, and discontinuation of lenvatinib

Side Effects	Severity	Procedures
Hypertension	Systolic pressure ≥ 140 mmHg and diastolic pressure ≥ 90 mmHg	Continue using lenvatinib in combination with antihypertensive agents
	Systolic pressure ≥ 160 mmHg and diastolic pressure ≥ 100 mmHg despite antihypertensive treatment	Withdraw until systolic pressure ≤ 150 mmHg or diastolic pressure ≤ 95 mmHg is achieved, followed by use of antihypertensive agents and resumption of lenvatinib treatment with a 1-stage reduction in dosage
	Side effects (grade 4)	Discontinuation of lenvatinib
Other side effects	Side effects (grade 2 or 3)	Withdraw lenvatinib until baseline status is regained or the condition is ameliorated to grade ≤ 1. If resumed, then lenvatinib should be administered with a 1-stage reduction in dosage
	Side effects (grade 4)	Discontinuation of lenvatinib

The side effect grade is based on the Common Terminology Criteria for Adverse Events (CTCAE; version 4.0). Grade 1: mild, with no symptoms or mild symptoms. Only clinical examination or laboratory testing is performed. Treatment is not required. Grade 2: moderate. Minimum/local/non-invasive treatments are required. Age-appropriate limitations of the activities of daily living, with the exception of personal commitments, are required as well. Grade 3: severe or clinically serious, no immediate threat to life, cannot be active/inoperable. Hospitalization or prolonged length of hospital stay is required. Limitations of the activities of daily living including personal commitments are required as well. Grade 4: life-threatening. Emergency treatment is required

The 2016 clinical practice guidelines for the management of kidney disease among cancer survivors [53] recommend that clinicians should individually evaluate the advantages and disadvantages of continuing the administration of VEGF inhibitors for patients who develop proteinuria and have practical treatment options for drug reduction or withdrawal while considering the patient’s requests. Although it is generally considered that renal damage secondary to lenvatinib treatment can be ameliorated with drug withdrawal, our findings indicated that lenvatinib can cause intractable and irreversible renal damage.

In conclusion, we report, for the first time, a case of lenvatinib-induced FSGS that resulted in irreversible renal damage. Lenvatinib is an antineoplastic agent used to treat thyroid cancer due to its inhibitory effects on several tyrosine kinases involved in tumor vascularization and growth. However, lenvatinib increases the risk of hypertension and proteinuria, which can result in nephrotic syndrome and acute renal damage. Our patient’s renal biopsy results confirmed the presence of FSGS caused by vascular endothelial disorder and podocyte damage due to TKI, which resulted in the development of complete hyalinization lesions and led to chronic kidney disease. Lenvatinib is a TKI with novel binding ability, but its renal damage mechanism seems to be similar to that of conventional TKIs. Moreover, the clinical course indicated that nephrotic syndrome remission was reached over the course of a protracted timeline after discontinuation of lenvatinib treatment. Ongoing screening for hypertension and proteinuria might be beneficial for early identification of possible renal damage in patients treated with lenvatinib. Based on our findings, we suggest that potential renal damage should be fully considered when using lenvatinib.

## Abbreviations

Alb: Albumin
CKD: Chronic kidney disease
c-MIP: c-maf-inducing protein
CR: Creatinine
CT: Computed tomography
eGFR: Estimated glomerular filtration rate
ET-1: Endothelin-1
fms: Feline McDonough sarcoma
FSGS: Focal segmental glomerulosclerosis
LDL: Low-density lipoprotein
NO: Nitric oxide
PAM: Periodic acid methenamine silver
PDGFRα: Platelet-derived factor receptor-α
PGI_2_: Production of prostacyclin
RET: Rearranged during transfection
sFlt1: Feline McDonough sarcoma virus-like tyrosine kinase-1
TC: Total creatinine
TDM: Therapeutic drug monitoring
TMA: Thrombotic microangiopathy
TP: Total protein
VEGFR: Vascular endothelial growth factor receptor
